# Thiamine antagonists trigger p53-dependent apoptosis in differentiated SH-SY5Y cells

**DOI:** 10.1038/s41598-017-10878-x

**Published:** 2017-09-06

**Authors:** Sergiy Chornyy, Yulia Parkhomenko, Nataliya Chorna

**Affiliations:** 1 Department of Psychology, University of Haifa, Haifa, Israel; 2 Department of Vitamin and Coenzyme Biochemistry, Palladine Institute of Biochemistry, Kyiv, Ukraine; 3 Department of Biochemistry, University of Puerto Rico, School of Medicine, San Juan, PR USA; 4 PR-INBRE Metabolomics Research Core, University of Puerto Rico, School of Medicine, San Juan, PR USA

## Abstract

Accumulating evidences suggest that p53 is a key coordinator of cellular events triggered by oxidative stress often associated with the impairment in thiamine metabolism and its functions. However, there are limited data regarding the pursuant feedback between p53 transactivation and thiamine homeostasis. Impairment in thiamine metabolism can be induced experimentally via interference with the thiamine uptake and/or inhibition of the thiamin pyrophosphate–dependent enzymes using thiamine antagonists - amprolium (AM), oxythiamine (OT) or pyrithiamine (PT). We found that exposure of neuronally differentiated SH-SY5Y cells to AM, OT and PT triggered upregulation of p53 gene expression, post-translational modification of p53 via phosphorylation and activation of p53 DNA-binding activity. Phosphorylation of p53 at Ser20 was equally efficient in upregulation of thiamine transporter 1 (THTR1) by all antagonists. However, induction of the expressions of the pyruvate dehydrogenase E1 component subunit beta (PDHB) and oxoglutarate dehydrogenase (OGDH) required dual phosphorylation of p53 at Ser9 and Ser20, seen in cells treated with PT and OT. Moreover, pretreatment of the cells with a decoy oligonucleotide carrying wild-type p53-response element markedly attenuated OT-induced THTR1, PDHB and OGDH gene expression suggesting an important role of p53 in transactivation of these genes. Finally, analysis of gene and metabolic networks showed that OT triggers cell apoptosis through the p53-dependent intrinsic pathway.

## Introduction

A large number of studies have shown that nerve cells are the most susceptible to a disturbance in thiamine homeostasis^1^. Inflammation, gliosis and variations in oxidative metabolism often accociated with reductions in thiamine linked processes result in DNA damage and neuronal cells death, which are thought to be the basis for many neurodegenerative diseases^2, 3^. It is recognized that cells sustaining the high level of DNA damage trigger activation of transcriptional factor p53 via post-translational modifications that provides the opportunities for apoptotic signal amplification^4^. However, evidences also suggest that p53 may be involved in DNA repair via preventing the replication of damaged DNA and activation of the expression of genes important for the protection of the cell from oxidative damage and molecules that are critical to thiamine homeostasis. Thus, it was shown that p53 triggers transactivation of thiamine transporter 1 (THTR-1) gene expression leading to an increase in thiamine uptake. Accumulated thiamine and its active form - thiamine pyrophosphate (TPP) are further implicated in negative feedback mechanisms that suppress intracellular p53 activity^5, 6^. This is especially important for the alleviation of the deleterious effects of mitochondrial dysfunction and allowing cells temporarily arrested in growth reenter to the cell cycle. While growing body of evidence suggests a critical function of p53 in thiamine homeostasis, the role of p53 in a coordination of cellular events triggered by changes in thiamine metabolism are not completely elucidated.

It is recognized that thiamine antagonists - amprolium (AM), oxythiamine (OT) and pyrithiamine (PT) metabolically interfere with thiamine metabolism and its functions^7^. All antagonists competitively inhibit thiamine transport while PT was found to be more efficient^8^. OT and PT, as competitive substrates of thiamine pyrophosphokinase (TPK), can restrict the level of TPP and at the same time become pyrophosphorylated (PP) by TPK. Moreover, according to current evidence, PT-PP could efficiently block TPK while OT-PP could compete with TPP coenzyme function and inhibit TPP-dependent enzymes leading to an impairment in thiamine homeostasis^7, 9–12^.

We previously showed that culturing of rat PC-12 and human SH-SY5Y cell lines differentiated into neurons^13, 14^ in the presence of 10 μM thiamine and different concentrations of PT, OT and AM dramatically decreased the cell viability in time and dose-dependent manner, triggered DNA fragmentation and resulted in stimulation of apoptosis via Caspase 3 - mediated signaling pathway. Notably, the cells appeared to be mostly vulnerable to PT and OT with the less extent to AM. This observation suggested that cells with neuronal phenotype treated with AM were still able to retain a low intracellular concentration of thiamine and undergo death slower than after treatment with OT or PT^13, 14^.

In the current study, we prompted to identify the effects of thiamine antagonists AM, OT and PT on the level of p53 gene expression, its post-translational modification, DNA-binding activity and its role in the regulation of metabolic and signaling pathways using RA-differentiated SH-SY5Y cells which are a widely-used model of human neurons. The recent studies showed that RA-differentiated SH-SY5Y cells exhibit a greater stimulation of mitochondrial respiration with uncoupling and an increased bioenergetic reserve capacity than undifferentiated^15^ and they represent a suitable experimental model to study the mechanisms of the oxidative stress in neuronal cells^16^. Moreover, SH-SY5Y cells express wild-type p53 protein that retains its functional apoptotic activity in response to DNA damage^17^. In addition, we explored the possible association between p53 and proteins involved in metabolism and functioning of thiamine such as THTR1 and thiamine-dependent enzymes: the pyruvate dehydrogenase E1 component subunit beta (PDHB), oxoglutarate dehydrogenase (OGDH) and transketolase (TK).

## Results and Discussion

### Effect of thiamine antagonists on the state of p53 protein in neuronal SH-SY5Y cells

p53 is recognized as a key coordinator of cellular events triggered by oxidative stress often associated with abnormalities in thiamine-dependent processes^18^. Moreover, depending on the stress level, p53 could exhibit pro-oxidative activities leading to cell death^19^. Our previous studies showed that a disturbance in thiamine homeostasis induced by AM, OT and PT triggered the Caspase 3 mediated apoptosis in nerve cells^13, 14^.

**Figure 1:**
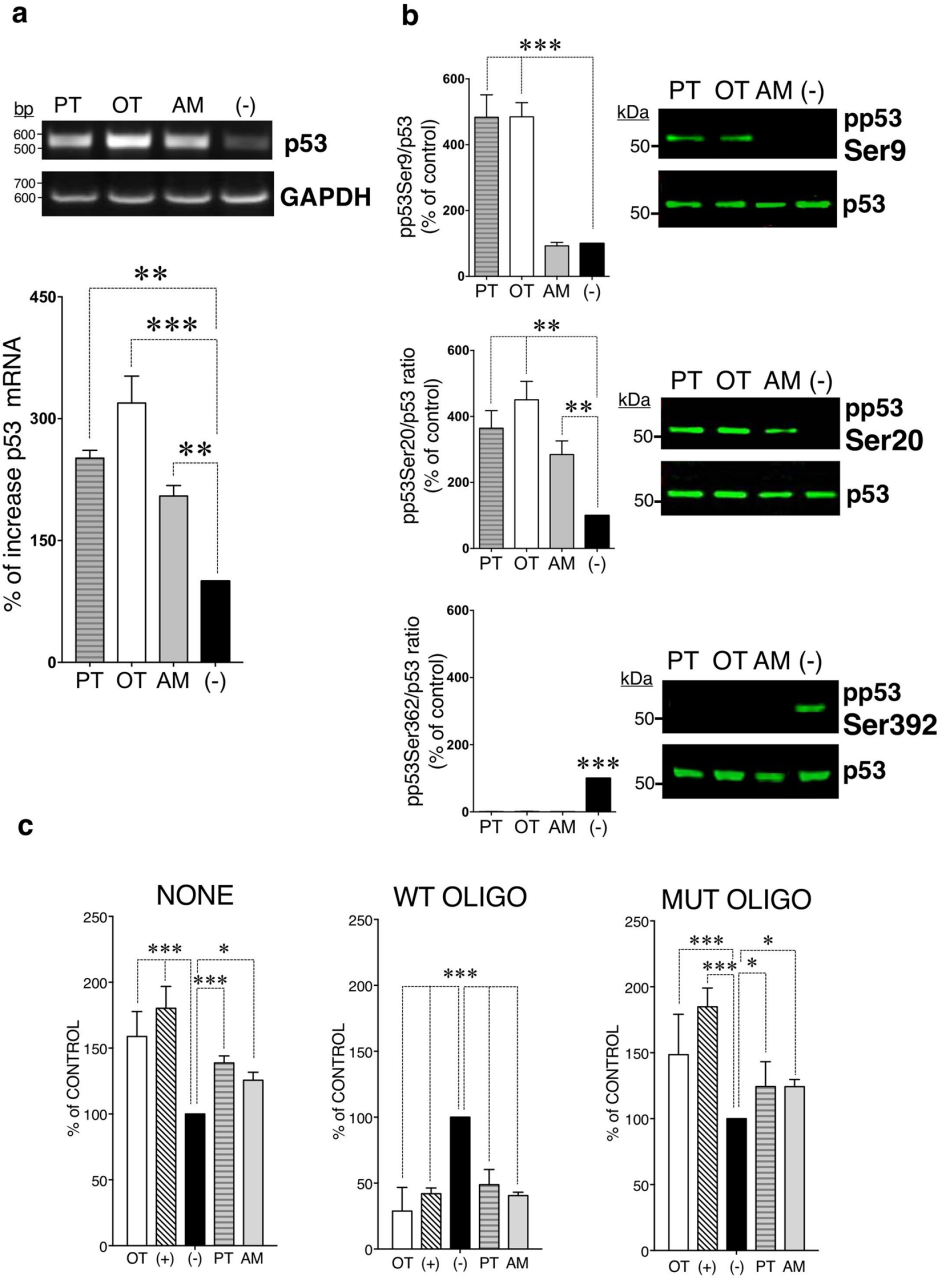
Effects of PT, OT and AM on the p53 gene expression, posttranslational modification and DNA-binding activity. Differentiated SH-SY5Y cells were treated with 100 μM PT, OT and AM. (**a)** p53 gene expression were determined by RT–PCR, normalized to GAPDH levels and mean ± SEM values (*n* = 3) were expressed as a percentage of the response to non-treated cells (−). (**b)** Western blots of phosphorylated p53 and total p53 were stained by appropriate antibodies. Data were normalized to total p53 and mean ± SEM values (*n* = 3) were expressed as a percentage of the response to non-treated cells (−). (**c)** The DNA-activity of p53 was determined by TransAM p53-DNA kit using nuclear extracts from cells treated with PT, OT, AM (NONE). Competitive binding experiments were performed by capturing of active p53 in nuclear extracts by wild type (WT OLIGO) or mutated oligonucleotide (MUT OLIGO) prior the binding assay. Non-treated cells were examined as negative control (−). MCF-7 nuclear cell extract treated with H_2_O_2_, provided with the TransAM kit, was used as a positive control (+). Data were calculated as percent of non-treated cells where the mean of its value was set to 100% (n = 3). Statistical analyses were performed using One way ANOVA (**a**,**b**) and Two-way RM ANOVA (**c**) followed by Bonferonni post-testing (*P < 0.05, **P < 0.01, ***P < 0.001).

Since there is a lack of evidences that suggest the involvement of p53 in regulation of apoptosis potentiated by thiamine antagonists, we prompted to study their effects on the p53 gene expression, posttranslational modifications and DNA-binding activity. A One-Way ANOVA analysis followed by Bonferroni’s multiple comparison test identified, that treatment of cells with 100 μM PT (251.3% ± 9.6, *P* < 0.01), OT (319.2% ± 33.2, *P* < 0.001) and AM (204.9% ± 12.8, *P* < 0.01) significantly increased the p53 gene expression while the most powerful effect was observed after treatment with OT (Fig. 1a).

It is known that the p53 protein exerts its functional diversity ranging from activation of cell death to cell survival mechanisms via specific post-translational modifications including phosphorylation, acetylation, mono- and di-methylation, glycosylation, ubiquitylation, neddylation, sumolyation, and poly-ribosylation at different amino acid residues^20^. p53 protein contains 393 amino acids that form several structural and functional domains^20^. In response to DNA damage and other cellular stresses^21^, the protein levels of p53 are significantly increased, and p53 acts as a transcription factor that regulates numerous of target genes^22^. Here we show, that 100 μM PT and OT induced dual p53 phosphorylation at Ser9 (PT: 364% ± 54, *P* < 0.01; OT: 451% ± 56, *P* < 0.001) and Ser20 (PT: 484% ± 68, *P* < 0.001; OT: 485% ± 43, *P* < 0.001). In contrast, 100 μM AM triggered phosphorylation only of p53 at Ser20 (AM: 344% ± 28, *P* < 0.01) (Fig. 1b). It is recognized that p53-induced apoptosis correlates with its phosphorylation at Ser9 and Ser20 in the transactivation domain 1 (residues 1–40), located at its N-terminus^23^. In particular, Ser20 triggers uncoupling the p53/MDM2 interaction^24^ while both Ser9 and Ser20 are important for stabilization of p53 transcriptional activity that couples to apoptotic signaling^25^. These data are in agreement with previously published that neuronal SH-SY5Y cells were more vulnerable to PT and OT in triggering apoptotic response compare to AM^14^. Furthermore, the carboxy-terminal phosphorylation of p53 at Ser392 occurring usually under normal unstressed conditions^26^ was detected exclusively in control cells (*P* < 0.001) (Fig. 1b).

We next examined whether p53 phosphorylation influenced on the transcriptional activity of p53. As determined by two-way RM ANOVA, 100 μM PT, OT and AM were effective in binding of p53 to oligonucleotide containing its consensus sequence (*P* = 0.4483), compare to non-treated control cells (***P < 0.001). These results suggest that the phosphorylation of p53 at Ser20 was sufficient to trigger p53 binding to DNA. However, the most significant effect was observed after treatment of the cells with OT (***P < 0.001) (Fig. 1c).

### Role of p53 in the expression of proteins involved in metabolism and functioning of thiamine in neuronal cells

**Figure 2:**
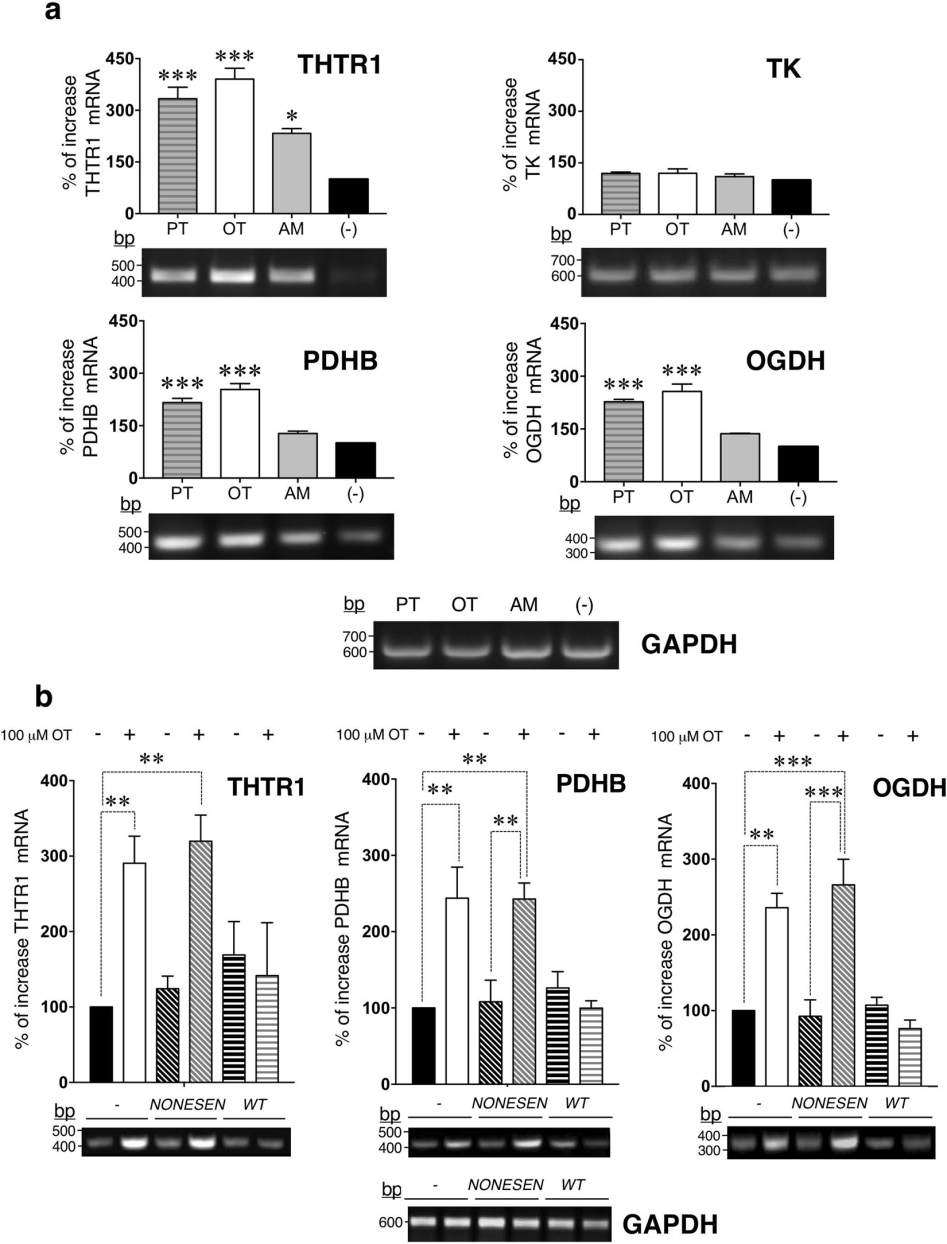
p53-dependent regulation of the expression of proteins involved in metabolism and functioning of thiamine by OT. Differentiated SH-SY5Y cells were treated with (**a)** 100 μM PT, OT and AM. THTR1, TK, PDHB, OGDH gene expression were determined by RT–PCR. (**b)** Differentiated SH-SY5Y cells were collected, electroporated with 1 μM of p53 decoy (wt) or nonsense oligonucleotide (nonsen) followed by treatment with 100 μM OT. THTR1, PDHB, OGDH gene expression were determined by RT–PCR. All presented data (**a**,**b**) were normalized to GAPDH and expressed as the percentages of increase over non-treated cells (−). One way ANOVA and Bonferroni post-testing (*P < 0.05, **P < 0.01, ***P < 0.001) indicates statistically significant differences between samples (n = 3).

There is limited evidence regarding pursuant feedback between p53 transactivation and thiamine homeostasis which suggested a direct regulation of THTR1 gene expression by p53^5, 6^. Analysis of THTR1 gene expression revealed that all antagonists significantly elevated the levels of THTR1 in SH-SY5Y cells (PT**:** 347% ± 21, *P* < 0.001; OT: 395% ± 28, *P* < 0.001 and AM: 232% ± 15, *P* < 0.01) (Fig. 2a). Elevation of THTR1 by thiamine antagonists might suggest an attempt of the cell to restore the required level of thiamine by increasing the number of the transporter molecules. We next evaluated the effects of PT, OT and AM treatments on the level of expression of three thiamine-dependent enzymes such as TK, a key enzyme of the pentose phosphate shunt, PDHC that links glycolysis and the tricarboxylic acid cycle (TCA) cycle, and OGDH, which is one of the rate-limiting steps in the TCA cycle^18^. We found that all antagonists had no influence on the expression of TK cells (PT: 119% ± 4, *P* = 0.26; OT: 120% ± 13, *P* = 0.22 and AM: 111% ± 7, *P* = 0.59) (Fig. 2a). However, analysis of the expression levels of PDHB and OGDH revealed that PT and OT were both effective in elevations of PDHB (PT**:** 193% ± 11, *P* < 0.001; OT: 258% ± 13, *P* < 0.001) and OGDH (PT: 209% ± 26, *P* < 0.05; OT: 256% ± 24, *P* < 0.01). In contrast, AM was not effective in modulation of PDHB (131% ± 9, *P* = 0.12) and OGDH (122% ± 16, *P* = 0.8) (Fig. 2a). Thus, upregulation of THTR1 gene expression by all antagonists correlates with p53 phosphorylation at Ser20 while upregulation of PDHB and OGDH required dual phosphorylation of p53 at Ser9 and Ser20, seen in cells treated with PT and OT (Figs 1b and 2a).

Taken together, our data suggest that interference with TPP coenzyme function by OT and PT is required for more pronounced p53 DNA-binding activity and as a result modulation of TPP-dependent enzymes expressions compare to interference with the thiamine uptake. OT and PT produced different physiological effects as seen in animal studies^10, 11^. Thus, administration of OT to animals produces primarily metabolic aberrations such as lethargy and anorexia while the administration of PT produces neurological problems similar to Wernicke-Korsakoff Syndrome^10^. However, the nature of these effects is still unknown. OT compare to PT could be more efficient in triggering an impairment of oxidative energy metabolism and the pentose-phosphate pathway^7, 12^. In addition, evidence suggested that OT can mimic non-coenzyme functions of thiamine affecting the activity of PDH and synthesis of acetylcholine from pyruvate^11^.

Since thiamine homeostasis is directly regulated by p53-mediated transactivation of the expression of THTR1 via interference with p53 binding to both the hdm2 and p21 DNA sites^5, 6^, the next experiments were performed, using OT as the most effective antagonist identified in our study. We prompted to establish if seen upregulations of THTR1, PDHB and OGDH in response to 100 μM OT were dependent on p53 transcriptional activation. Thus, we used oligo-decoy analysis to trap activated p53 and prevent its binding to DNA containing consensus sequence. We found that pre-treatment of the cells with p53 oligo-decoy displayed significant reduction in the expression of THTR1, PDHB, OGDH compare to nonsense oligonucleotide or non-treated controls (***P < 0.0001; two-way RM ANOVA) (Fig. 2b). Our data are in agreement with recently published study described the mitochondrial function of p53 in neurons^27^. Consequently, p53 not only activates PDC via downregulation of the expression of pyruvate dehydrogenase kinase 2 (PDK2), but also triggers upregulation of PDHB (Fig. 2b) – one of the components of PDC. Inhibition of the PDHB function by OT decreases the oxidation of pyruvate in mitochondria which facilitates the flux of pyruvate to lactate rather than to Acetyl-CoA^28^. Indeed, the analysis of metabolites showed the increase of lactate level (Supplementary Table S1).

### Networks analyses

**Figure 3:**
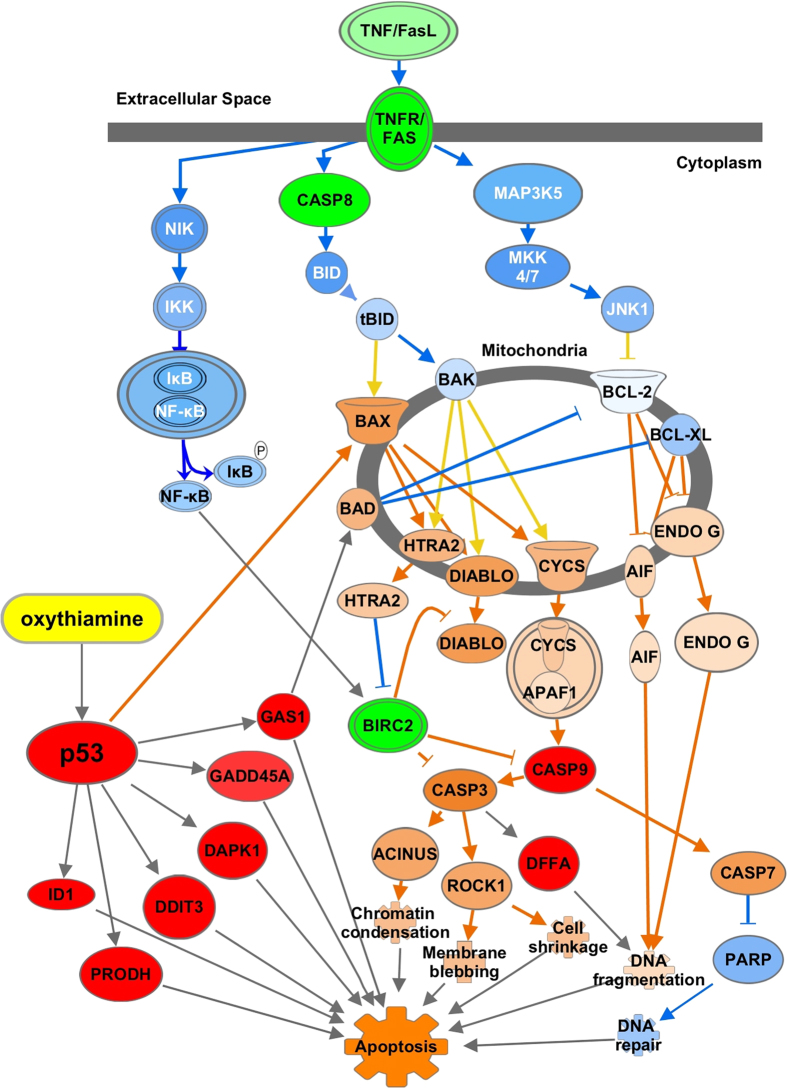
IPA canonical pathway analysis and molecular activity of genes regulated by p53 in response to OT in differentiated SH-SY5Y cells. Red denotes detected upregulation, green - detected downregulation. The molecular activity prediction tool was used to simulate the effect on p53 on gene expression. Correspondently, orange showed predicted upregulation, and blue - predicted downregulation. The pathway reconstructed using IPA pathway builde.

Ingenuity Pathway Analysis (IPA) was used to analyse, integrate, and understand data of the differentially expressed genes and metabolites triggered by the most effective thiamine antagonist - OT at a higher dose equal 1000 μM for 72 h. A metabolic profiling approach using gas chromatography coupled to mass spectrometry was chosen. For gene expression analysis, we used a cDNA array containing genes of apoptotic interest to determine potential gene targets. We identified 26 metabolites (Supplementary Table S1) and 40 genes (Supplementary Table S2) that were mapped to the Ingenuity Knowledge Database. Thus, p53 was identified as the top upstream regulator (*P* = 1.50E-12) and “Pro-Apoptosis” (*P* = 3.18E-17) as the top of predicted molecular and cellular functions. An additional functional analysis was conducted using 26 identified metabolites mapped together with genes of proteins related to thiamine metabolism including PDHB, OGDH and p53 identified by RT-PCR. The predicted biological functions associated with OT treatment were (1) triggering of oxidative stress (*P* = 2.46E-03), loss of mitochondrial membrane potential (*P* = 2.30E-02), which in turn activates the mitochondrial permeability transition (MPT) pore (*P* = 9.00E-03) releasing of both NADH and cytochrome C into the cytoplasm and thereby affecting cellular respiration, triggering mitochondrial dysfunction and apoptotic cell death^29^. These events could also be potentiated by OT-dependent accumulation of p53, which upon activation translocates inside of the mitochondria and might physically interact with pro-apoptotic members (Bak, Bax) of the Bcl2 family^30^. Impairment in cellular respiration often triggers swelling of the mitochondria (*P* = 1.92E-02). Moreover, these effects were exacerbated after inhibitions of OGDH and PDHB by OT that led to energy failure due to dysfunction of the TCA cycle flux (*P* = 1.81E-09). In addition, we identified an accumulation of isocitrate after treatment of SH-SY5Y cells with OT (Supplementary Table S1). It is recognized, that inhibition of OGDH activity may trigger the α-ketoglutarate conversion to isocitrate that may result in a TCA cycle functioning in a reverse mode^31^. In this case, the levels of succinate, fumarate and malate are expected to be decreased as seen in our experiment (Supplementary Table S1). In addition, we found an elevation of the content of the fatty acids i.e. myristate, palmitate, stearate, palmitoleate and oleate (Supplementary Table S1) that possibly results from a disrupting of the membranes integrity by OT^13, 14^. Furthermore, we identified disturbances in amino acids homeostasis as reflected by a reduction in the content of glycogenic amino acids pool - alanine, glycine, proline, aspartate and glutamate, and both glycogenic and ketogenic amino acids pool – phenylalanine, tyrosine and threonine. Down-regulation of glutamate together with, 5-oxoproline, glycine and proline indicates dysfunction of the γ-glutamyl cycle (*P* = 1.56E-03) and proline metabolism (*P* = 3.39E-03) (Supplementary Table S1). Furthermore, OT significantly induces the expression of the PRODH (Supplementary Table S2; Fig. 3), a mitochondrial enzyme, known to be transcriptionally activated by p53^32^. PRODH catalyzes the first step in proline degradation via its conversation to pyrroline-5-carboxylate (P5C) thereby shuttling redox between cytosol and mitochondria with the concomitant transfer of electrons to cytochrome C and activation of caspases^33^. Identified reduction in the level of proline by treatment suggests that it is not converted back to P5C and mostly utilized through formation of proline-dependent reactive oxygen species^33^. Moreover, inhibition of PDHB by OT led to a decrease of the nervous system-specific metabolite *N*-acetyl-aspartate (Supplementary Table S1), which is synthesized from aspartate and acetyl-coenzyme A in neurons, and is a key metabolite in many biochemical features of CNS development and function^34^.

It is known that the activation of apoptosis can be triggered either by the intrinsic (mitochondrial mediated) or extrinsic (death receptor mediated) apoptotic pathways^35^. We found that genes associated with the Death Receptors Signaling Network such as members of tumor necrosis factor receptors superfamily (TNFRS): TNFRSF1A, TNFRSF1B, TNFRSF10B, TNFRSF25, as well as the other components of the death receptor signaling pathway including FASLG, FASTK and Caspase 8 were significantly downregulated (Fig. 3). IPA-MAP enabled us to analyze the regulation of the downstream effects of in the Death Receptors Signaling Network which suggests that OT does not affect this pathway. Thus, NF-κB-inducing kinase (NIK) – the direct downstream effector of the TNFRSFs^36^ was predicted to be downregulated affecting the signaling to IKK complex kinases and as the result the activation of NF-κB^37^ (Fig. 3). Moreover, downregulation of Caspase 8 is expected to decrease the activation of pro-apoptotic factors BID and BAX^38^.

In accordance to previous experiments (Fig. 1a), we observed a significant upregulation of transcription factors DDIT3 and ID1, which are p53-target genes involved in apoptosis^39^ (Fig. 3). Furthermore, OT triggered upregulation of an essential stress sensor GADD45A, which is mediated by both p53-dependent and independent mechanisms. It is known that GADD45A can coordinate either cell survival or cell arrest via physical interaction with partner proteins either PCNA, p21, cdc2/cyclinB1, or the p38 and JNK stress response kinases^40^. Other genes implicated in the apoptotic pathway and upregulated by OT at different levels included those encoding Caspase 9, DAPK 1, DFFA and GAS1 (Fig. 3). It is recognized that GAS1 acts as a growth suppressor, blocking the cell cycle before S phase in a p53-dependent manner^41^. Further, in accordance with our results, recent studies of the role of GAS1 in triggering of apoptosis using SH-SY5Y cells have shown that serum deprivation triggered intrinsic apoptotic pathway including activation of BAD, release of cytochrome C and activation of Caspase 9 and Caspase 3^42^. Moreover, accordingly to IPA MAP tool, p53 can directly activate pro-apoptotic factor Bax^43^ and trigger a cascade of activation of other pro-apoptotic factors such as HTRA2 and DIABLO^44^, Cytochrome C release and apoptosome formation^45^, activation of AIF and ENDO G that couple to DNA fragmentation^46^.

Increase in Caspase 9 gene expression by OT as predicted by IPA can couple to cleavage of Caspase 3^47^ followed by activation of nuclear factor ACINUS required for apoptotic chromatin condensation^48^ and ROCK-1^49^ that contributes to the membrane blebbing and cell shrinkage^50^. Moreover, active Caspase 9 can increase activation of Caspase 7^51^ and leading to inhibition of PARP-dependent DNA repair^52^. Predicted activation of Caspase 3 by OT is in agreement with our previous results where we showed that thiamine antagonists PT, OT and AM trigger Caspase 3 cleavage and DNA fragmentation in neuronal cells^13, 14^. Moreover, observed increase in DFFA gene expression could also occur due to Caspase 3 activation leading to DNA fragmentation^53^. We also observed enhanced expression of RHOA – which upon accumulation in the cytoplasm activates Rho-associated protein kinase that inactivates Myosin light chain (MLC) phosphatase, leading to increased levels of phosphorylated MLC, which is involved in the membrane blebbing^54^.

Moreover, several genes implicated in cell survival mechanisms were significantly downregulated by OT including BIRC2, NME2, E2F3, and E2F5 (Supplementary Table S2, Fig. 3). Treatment with OT triggered differential expression of secreted frizzled related proteins (SFRP) 2 and 5 – known as soluble modulators of Wnt signaling (Supplementary Table S2). Thus, SFRP 2 was decreased while SFRP 5 significantly increased. It is recognized that SFRP 2 transduces anti-apoptosis signals via NF-κB activation^55^. This agrees with our previous conclusion that NF-κB signaling pathway remains unaffected in response to OT treatment. In contrast, SFRP 5 exerts an apoptotic function^56^.

Given the important role of p53 in coordination of cellular events triggered by oxidative stress as the result of the impairment in thiamine metabolism, we showed the possible association between p53 transactivation and thiamine homeostasis that results in triggering of p53-dependent intrinsic apoptotic pathway. To our knowledge, this is the first observation that shows a p53-dependent regulation of THTR-1, PDHB and OGDH gene expression in cellular model of experimentally induced interference with the thiamine uptake and/or inhibition of the thiamin pyrophosphate–dependent enzymes using thiamine antagonists. Understanding the role of thiamine and its antagonits in cell signaling processes important for both fundamental and practical points of view. Taken together, our results provide a new information of the specific action of thiamine antagonists on the signaling processes of apoptosis that can apply for the development of new experimental cellular models that can be used to study the molecular mechanisms of apoptosis, oxidative stress and thiamine metabolism and functions.

## Materials and Methods

### Cell culture

Human SH-SY5Y neuroblastoma cells (ATCC, USA) were cultured in Dulbecco’s Modified Eagle’s/F12 medium (DMEM/F12, Sigma-Aldrich), 10% FCIII (VWR) and 1% antibiotic-antimycotic mixture (Sigma-Aldrich) in 5% CO_2_ at 37 °C. In prior experiments, SH-SY5Y cells were incubated in a complete FCIII medium at a low serum content (1%) in presence of 15 μM RA (Sigma-Aldrich) for 72 h as previously described by us^14^ followed by treatment with PT, OT and AM (Sigma-Aldrich) for an additional 72 h using the concentrations described below. Cells incubated without thiamine antagonists were analyzed as control. Each experiment was repeated three times.

### Western Blot Analysis

Cells were seeded at a density of 1 × 10^6^ cells onto in six-well plates (Sigma-Aldrich) and incubated with 100 µM PT, OT and AM as above. After the incubation, the cells were washed with ice-cold PBS and processed as described by us^13^. Immunostaining of phosphorylated p53 at Ser9, Ser20, and Ser392 on nitrocellulose membranes (1:1000, GE Healthcare) was performed using the phospho-p53 antibody sampler kit (Cell Signaling Technology, 9919) followed by secondary detection using IRDye-800 goat anti-rabbit antibody (1:5000, Licor Biosciences, 925–32211) and visualized using the Licor Odyssey Imaging system (Licor Biosciences).

### RNA isolation

Cells were seeded at a density of 1 × 10^6^ cells onto in six-well plates (Sigma-Aldrich) and incubated with 100 µM PT, OT and AM as above. Total RNA was isolated using the TRIZOL^®^ reagent (Thermofisher Sci) followed by DNase I treatment (Quiagen) and processed as described by us^13^. The concentration and integrity of RNA were determined using the NanoDrop-1000 Spectrophotometer (Thermofisher Sci).

### RT–PCR

cDNA was obtained using the Reverse Transcription System kit (Promega, A3500) followed by PCR in a total volume of 25 µL PCR Master Mix (Promega, M7502) as previously described by us^13^ with 1 µM of the indicated sets of primers: human p53 (NM_000546.2; 5′-gtggaaggaaatttgcgtgt-3′ and 5′-tctgagtcaggcccttctgt-3′), human THTR1 (NM_006996.1; 5′-accccagcttctaaccacct-3′ and 5′-gtggaaggaaatttgcgtgt-3′), human OGDH (NM_001003941; 5′-ggaatcagcacttcctctgc-3′ and 5′-acgtagtccacgccattctc-3′), human PDHB (NM_000925; 5′-tccctggaattcagaggatg-3′ and 5′-agcaccagtgacacgaacag-3′), human TK (NM_001064; 5′-caaaaacatggctgagcaga-3′ and 5′-ttgtattggcggctagttcc-3′) and human GAPDH (A03911; 5′-tgaaggtcggactcaacggatttggt-3′ and 5′-gtggtggacctcatggcccacatg-3′). 5 μl PCR products were analyzed by 1% agarose gel electrophoresis. The bands obtained were stained with ethidium bromide (Sigma-Aldrich) and visualized under ultraviolet light using Versa Doc™ equipped with QuantityOne^©^ software (Bio-Rad).

### p53-DNA Binding Enzyme-linked Immunosorbent Assay

Nuclear cell extracts (10 μg) from cells treated with 100 µM PT, OT and AM were used to detect activation of the p53 by the TransAM p53 assay kit (Active Motif, 41196) following the instructions of the manufacturer. To validate the results of p53 activation by the thiamine antagonists, competitive binding experiments were performed by adding 20 pmol of wild type or mutated oligonucleotide, supplied by the kit, to nuclear cell extracts to capture active p53 prior the binding assay per the instructions of the manufacturer.

### Capturing of p53 with decoy oligonucleotides

Decoy oligonucleotides containing the palindromic p53 *cis*-element 5′-RRRCWWGYYY-3′ (R = A or G, Y = C or T, W = A or T), which allows self-hybridization and formation of a duplex hairpin that competes with p53 enhancers for binding of transcription factors, were used to inhibit p53-directed transcription *in vivo*, as previously described by us^57^. The sequences of the p53 decoy and control phosphorothioate oligonucleotides (underscored) (Sigma-Aldrich) were as follows: p53 decoy, 5′-GGACATGCCCGGGCATGTCC-3′; control nonsense sequence, 5′-CTAGCTAGCTAGCTAGCTAGCTAG-3′. Cells were seeded at a density of 10^6^ cells onto in 100 mm Petri dishes (Sigma-Aldrich), differentiated as above. After that cells were collected and 800  μl of 2.5 × 10^7^ cell/ml, electroporated in presence of 500 μg sheared salmon sperm DNA (Sigma-Aldrich) and 1 μM of each phosphorothioate oligonucleotide at 330 V and 1000 uF using an Electroporator II (Invitrogen). Next, cells were plated onto 6-well plates (Sigma-Aldrich), grown in a complete medium overnight followed by its replacement with 1% FCIII containing 100 μM OT for 72 h and analysis using RT-PCR as said above.

### RNA Preparation and cDNA array analysis

50 µg of the total RNA isolated from SH-SY5Y cells differentiated as above and treated with 1000 μM OT for 72 h were used to synthesize biotin-labeled probes with a pooled set of primers complementary to 205 human cDNAs spotted on the Atlas^TM^ Human cDNA Apoptosis Array (BD Biosciences Clontech, 7743–1) using the BD SpotLight^TM^ Labeling Kit (BD Biosciences Clontech, 634801). Probes were detected via labeling with IRDye-680RD-streptavidin (1:7000, Licor Biosciences, 926–68079). Signal visualization was performed using the Odyssey Infrared Imaging System (LICOR Biosciences). Analysis and quantification was conducted using the AtlasImage 2.0 software (BD Biosciences Clontech) and global normalization method (sum method). Genes that showed no expression values in one or more analyzed experimental groups (control *vs* treatment, n = 3) were excluded from further analysis. The gene expression responses were calculated as ratios between OT and control, and compared to the expression level of housekeeping gene (60 S ribosomal protein (L13A)). The cDNA array data described here have been deposited at the NCBI gene expression and hybridization array data repository (GEO): under Accession No. GSE93842.

### Metabolite extraction, detection and identification

Cells at a density of 3 × 10^6^ cells/ml were seed onto in 150 mm Petri dishes (Sigma-Aldrich), differentiated as above and treated with 1000 μM OT for an additional 72 h. Metabolites were extracted and derivatized as previously described by Dettmer *et al*.^58^. The analytes were fractionated by gas chromatography-mass spectrometry (GC-MS) (GCMS-QP2010, Shimadzu Scientific). The chromatography conditions were as follows: RXI-5MS (0.25 mm inner diameter, 0.25 μm D.F., 30 m) (Restek), split injection (ratio = 15), injection volume of 1 μL. Inlet temperature was 280 °C; ion source temperature was 200 °C; interface temperature was 150 °C. The oven temperature was set at 100 °C for 1 min, and then programmed from 100 °C to 290 °C at 8 °C/min, and held at 290 °C for 16 min. Helium was used as the carrier gas at a constant linear velocity of 39 cm/sec. MS conditions were set as follows: Electrospray ionization (ESI) source, full scan mode, electron energy of 70 eV, quadrupole scan range of m/z 35–700. Data were processed using the GCMS Solution Postrun Analysis software (Shimadzu Corp) for metabolites identification from their electron impact mass spectra by comparison to the NIST 2011 spectral mass library.

### Ingenuity Pathway Analysis

The functional analysis was conducted using the Ingenuity Pathway Analysis (IPA) core analysis of metabolites (QIAGEN). Data sets containing identifiers such as the Human Metabolome Database (HMDB) compound IDs or Gene Bank IDs and their corresponding experimental ratio values relatively to control converted by IPA to a fold change values were mapped to its corresponding object in the Ingenuity Knowledge Database (including canonical pathways and biological functions). Then, Fisher’s Exact algorithm calculated the probability of each metabolite’s set or gene’s set was enriched. Only the biological functions/pathways (Bonferroni’s corrected *P* value < 0.05) was considered significantly enriched with an overlap *P* value ≤ 1.3. The detailed descriptions of IPA analysis are available under “Ingenuity Canonical Pathways Analysis” and “Biological Functions Analysis”, and on the IPA website (http://www.ingenuity.com). Focus molecules were identified by IPA based on highest connectivity. Figure diagram was reconstructed using IPA pathway builder.

### Statistical analysis

Statistical analysis was performed with the Prism 7 software (GraphPad). All data are presented as percentage of the response to non-treated cells (mean) ± standard error of the mean (SEM). Statistical significance was assumed at *P < 0.05, **P < 0.01 or ***P < 0.001 as determined by a Student’s *t*-test for cDNA Array Analysis, and Ordinary One-way ANOVA or Two-way repeated measures ANOVA (two-way RM ANOVA) followed by Bonferroni test for multigroup comparisons in other experiments as indicated in the Results and Discussion section and figures Legends.

### Data Availability

The datasets generated during and analysed during the current study are available in the NCBI gene expression and hybridization array data repository (GEO): under Accession No. GSE93842. All other data generated or analysed during this study are available from the corresponding author on reasonable request.

## Electronic supplementary material

Supplementary Information
