# Supplementary material for: Thiamine antagonists trigger p53-dependent apoptosis in differentiated SH-SY5Y cells
Source: Sci Rep. 2017 Sep 6;7:10632. doi: 10.1038/s41598-017-10878-x (PMC5587765; doi:10.1038/s41598-017-10878-x)
Supplement: Supplementary file 1 — Supplementary Information [file 41598_2017_10878_MOESM1_ESM.pdf]

Thiamine antagonists trigger p53-dependent apoptosis in differentiated SH-SY5Y cells

Sergiy Chornyy[1], Yulia Parkhomenko[2] and Nataliya Chorna[3,4]

[1]Department of Psychology, University of Haifa, Haifa, IL

[2] Department of Vitamin and Coenzyme Biochemistry, Palladine Institute of Biochemistry, Kyiv, UA

[3]Department of Biochemistry, University of Puerto Rico, School of Medicine, San Juan, PR, USA

[4]PR-INBRE Metabolomics Research Core, University of Puerto Rico, School of Medicine, San Juan, PR, USA

Correspondence should be addressed to Dr. Nataliya Chorna, Department of Biochemistry, University of Puerto Rico, School of Medicine, A632 Main Building, San Juan PR 00935; e-mail: nataliya.chorna@upr.edu, Ph: 787-758-2525 ext. 1640

Supplementary Table S1 Metabolites content in neuronally differentiated SH-SY5Y after exposure to 1000 μM OT for 72 h.

N	HMDB ID[a]	NAME	RT (min)[b]	FOLD CHANGE[c]	CONTENT	CHARACTERISTIC FRAGMENT IONS
**1**	HMDB00190	L-lactic acid	9.89	1.24	up	73,147,261
**2**	HMDB00042	Acetic acid	10.5	1.11		73,147,189
**3**	HMDB00161	L-alanine	10.99	-2.02	down	73,158,232
**4**	HMDB00123	Glycine	11.31	-1.70	down	73,147,218
**5**	HMDB00883	L-valine	12.73	1.01		73,186,260
**6**	HMDB00687	L-leucine	13.3	1.08		73, 200, 274
**7**	HMDB00172	L-isoleucine	13.74	1.06		73, 200, 274
**8**	HMDB00254	Succinic acid	14.1	-1.13	down	73,147,289
**9**	HMDB00162	L-proline	14.25	-2.90	down	73,184,258
**10**	HMDB00134	Fumaric acid	14.4	-1.87	down	73,287
**11**	HMDB00267	5-Oxoproline	16.21	-1.39	down	73,272,300
**12**	HMDB00167	L-threonine	17.09	-2.02	down	73,303
**13**	HMDB00806	Myristic acid	17.77	1.70	up	75,285
**14**	HMDB00159	L-phenylalanine	17.89	-1.61	down	73,234,302
**15**	HMDB00812	N-acetyl-L-aspartic acid	18	-1.29	down	73,346
**16**	HMDB00156	Malic acid	18.12	-1.82	down	73,115,419
**17**	HMDB00191	L-aspartic acid	19.03	-1.38	down	73,302,418
**18**	HMDB03229	Palmitoleic acid	20.24	1.36	up	75,129,311
**19**	HMDB00148	L-glutamic acid	20.32	-1.54	down	73,272,432
**20**	HMDB00220	Palmitic acid	20.43	1.35	up	75,129,313
**21**	HMDB00207	Oleic acid	22.34	1.36	up	75,129,339
**22**	HMDB00827	Stearic acid	22.5	1.27	up	75,129,341
**23**	HMDB00094	Citric acid	23.84	1.17		73,459,591
**24**	HMDB00158	L-tyrosine	24.04	-1.08	down	73,302
**25**	HMDB01874	Isocitric acid	24.59	1.32	up	73,345
**26**	HMDB00067	Cholesterol	37.2	-1.30	down	75,443

[a] Human Metabolome Database (HMDB) compound IDs
[b] Retention time
[c] Metabolite fold changes were computed by Ingenuity Pathway Analysis (Quiagen) from experimental ratio values calculated using average peak intensity of each metabolite (OT vs non-treated control cells) obtained from the GCMS Solution Postrun Analysis software (Shimadzu Corp).

Supplementary Table S2 Genes differentially regulated in neuronally differentiated SH-SY5Y cells after exposure to 1000 μM OT for 72 h

N	GENBANK	SYMBOL	DESCRIPTION	Average[a] Ratio OT/(-)	EXPRESSION	*P* value[b]
**1**	X56932	RPL13A	Ribosomal protein L13a (Houskeeping gene)	1.03		
**2**	NP_002039.2	GAS1	Growth arrest specific 1 protein	1.76	up	$P < 0.01$
**3**	U60520	CASP8	Caspase 8, apoptosis-related cysteine peptidase	0.69	down	$P < 0.05$
**4**	U56390	CASP9	Caspase 9, apoptosis-related cysteine peptidase	1.85	up	$P < 0.01$
**5**	U60519	CASP10	Caspase 10, apoptosis-related cysteine peptidase	1.06		
**6**	U21092	TRAF3	CD40 receptor-associated factor 1 (CRAF1)	1.40		
**7**	NP_001243092.1	BIRC2	Baculoviral IAP repeat containing 2	0.63	down	$P < 0.01$
**8**	D38122	FASLG	Fas ligand (TNF superfamily, member 6)	0.43	down	$P < 0.05$
**9**	AF017986	SFRP2	Secreted frizzled-related protein 2	0.81	down	$P < 0.05$
**10**	AF017988	SFRP5	Secreted frizzled-related protein 5	2.25	up	$P < 0.01$
**11**	M33294	TNFRSF1A	Tumor necrosis factor receptor superfamily, member 1A	0.55	down	$P < 0.01$
**12**	M32315	TNFRSF1B	Tumor necrosis factor receptor superfamily, member 1B	0.68	down	$P < 0.05$
**13**	AF016268	TNFRSF10B	Tumor necrosis factor receptor superfamily, member 10b	0.73	down	$P < 0.01$
**14**	Y09392	TNFRSF25	Tumor necrosis factor receptor superfamily, member 25	0.85		$P < 0.05$
**15**	M27544	IGF1	Insulin-like growth factor 1 (somatomedin C)	1.02		
**16**	M35410	IGFBP2	Insulin-like growth factor binding protein 2, 36kDa	1.24		
**17**	M31159	IGFBP3	Insulin-like growth factor binding protein 3	0.77		

**18**	M62402	IGFBP6	Insulin-like growth factor binding protein 6	0.94		
**19**	X76104	DAPK1	Death-associated protein kinase 1	2.32	up	*P* < 0.001
**20**	X86779	FASTK	Fas-activated serine/threonine kinase	0.37	down	*P* < 0.05
**21**	U91985	DFFA	DNA fragmentation factor 45 (DFF45)	2.10	up	*P* < 0.01
**22**	AF022385	PDCD10	Apoptosis-related protein TFAR15	2.63	up	*P* < 0.05
**23**	NP_001655.1	RHOA	Ras homolog family member A	3.08	up	*P* < 0.01
**24**	D13889	ID1	DNA-binding protein inhibitor ID-1; Id-1H	3.23	up	*P* < 0.05
**25**	NP_001181982.1	DDIT3	DNA-damage-inducible transcript 3 (DDIT3)	2.49	up	*P* < 0.001
**26**	M60974	GADD45A	Growth arrest and DNA-damage-inducible, alpha	1.83	up	*P* < 0.05
**27**	M84820	RXRB	Retinoid X receptor, beta	1.04		
**28**	X07282	RARB	Retinoic acid receptor, beta	1.05		
**29**	L20320	CDK7	Cyclin-dependent kinase 7	1.07		
**30**	NP_004926.1	CDK5	Cyclin-dependent-like kinase 5 isoform 1	1.04		
**31**	L29220	CLK3	CDC-like kinase 3 (CLK3)	1.03		
**32**	X66362	CDK18	Cyclin-dependent kinase 18	1.49		
**33**	L22005	CDC34	Cell division cycle 34	0.65		
**34**	NP_620407.1	MAPK1	Mitogen-activated protein kinase 1	0.79		
**35**	U25278	MAPK7	Mitogen-activated protein kinase 7	1.26		
**36**	Y10479	E2F3	Transcription factor E2F3	0.54	down	*P* < 0.05
**37**	U15642	E2F5	Transcription factor E2F5	0.37	down	*P* < 0.05
**38**	U18422	TFDP2	Transcription factor DP2 (Humdp2); E2F dimerization partner 2	1.23		
**39**	M36981	NME2	NME/NM23 nucleoside diphosphate kinase 2	0.80	down	*P* < 0.05
**40**	AF010311	PRODH	proline dehydrogenase 1	2.42	up	*P* < 0.05

[a] Analysis and quantification was conducted using the AtlasImage 2.0 software (BD Biosciences Clontech) and global normalization method (sum method) as said in Material and methods section. The gene expression responses were calculated as ratios between adjusted intensities obtained for OT and control non-treated (-) cells.

[b] The *P* values were assessed using ratios calculated for the individual genes compare to housekeeping gene (60S ribosomal protein (L13A)) using Student's *t*-test. Statistical significance was assumed at *$P < 0.05$, **$P < 0.01$ or ***$P < 0.001$
